# Fistulized multidrug-resistant tuberculosis

**DOI:** 10.11604/pamj.2017.26.67.11745

**Published:** 2017-02-05

**Authors:** Naoual El Omri, Fadwa Mekouar

**Affiliations:** 1 Internal Medicine Department, Mohammed V Military Teaching Hospital, Rabat, Morocco

**Keywords:** Tuberculosis, multiresistant, fistula

## Image in medicine

A 21-year-old woman with no history of tuberculosis was admitted to the emergency room with an acute abdominal accompanied by fever. She underwent an emergency abdominal operation surgery and histological studies revealed a peritoneal and appendicular ovarian tuberculosis. The patient received antibacillar (Rifampicine, Isoniazid, Pyrazinamide). Two months later, she was readmitted with fever and a general physical deterioration while being on antituberculosis medications. Chest X-Ray and CT scan revealed a miliary tuberculosis with pleural and peritoneal effusion and lymphadenopathy above and below the diaphragm. HIV serology was negative. This peritoneal collection had caused a fistula on the abdominal wall with two sinus tracts in the midline. The bacteriological examination of the purulent material revealed a Mycobacterium tuberculosis with a high resistance to all anti-bacillar drugs except Etambuthol and Pyrazinamide. The patient was treated by etionamide, pyrazinamide, spiramycin and etambutole, with favorable outcome.

**Figure 1:**
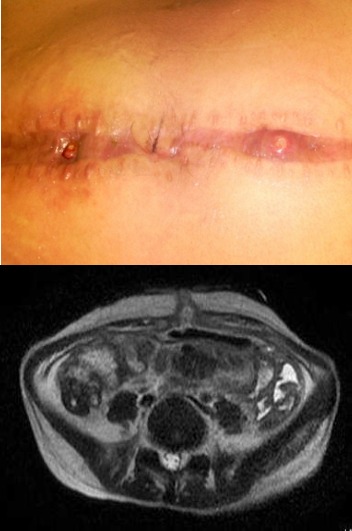
Two sinus tracts in the midline of abdominal wall and abdominal computed tomography showing the sinus tract

